# Autism and COVID-19: A Case Series in a Neurodevelopmental Unit

**DOI:** 10.3390/jcm9092937

**Published:** 2020-09-11

**Authors:** Leslie Nollace, Cora Cravero, Alice Abbou, Brice Mazda-Walter, Alexandre Bleibtreu, Nathalie Pereirra, Myriam Sainte-Marie, David Cohen, Marianna Giannitelli

**Affiliations:** 1 Department of Child and Adolescent Psychiatry, Reference Centre for Rare Psychiatric Diseases, APHP. Sorbonne Université, 75013 Paris, France; leslie.nollace@aphp.fr (L.N.); cora.cravero@aphp.fr (C.C.); alice.abbou@aphp.fr (A.A.); BRICE.MAZDA-WALTER@aphp.fr (B.M.-W.); nathalie.pereirra@aphp.fr (N.P.); myriam.saintemarie@aphp.fr (M.S.-M.); marianna.giannitelli@aphp.fr (M.G.); 2 Interdepartmental Mobile Unit for Complex Situations in Autism (UMI 75-92), Elan Retrouvé Foundation, 75009 Paris, France; 3 Department of Infectious Diseases, Pitié-Salpêtrière Hospital, APHP. Sorbonne Université, 75013 Paris, France; alexandre.bleibtreu@aphp.fr; 4 Institut des Systèmes Intelligents et Robotiques (ISIR), CNRS UMR 7222, Sorbonne Université, 75005 Paris, France

**Keywords:** COVID-19, autism, intellectual disability, challenging behaviors

## Abstract

Background: COVID-19 has become pandemic and can impact individuals with autism as well. Here, we report a case series admitted to a neurobehavioral unit dedicated to challenging behaviors in patients with autism. Methods: We describe 16 patients (mean age 20.8 years; range 12–43 years; 76% male) with autism hospitalized between March 2020 and mid-April 2020 for challenging behaviors, for which COVID-19 disease has been suspected and who needed both psychiatric and medical care. A close cooperation with the Infectious and Tropical Diseases Department was organized to limit viral spread and training sessions (e.g., hygiene, clinical COVID-19 monitoring, virus testing) were given to staff members. Results: Most patients had severe autism and severe/moderate intellectual disability. Eleven patients were already in the unit when it was hit by the pandemic, and five were admitted from the community. Based on a virus search via reverse transcriptase polymerase chain reaction (RT-PCR) or serology at the 2-month follow-up, we had 11 confirmed COVID-19 cases. The main COVID-19 symptoms included benign upper respiratory infection signs (*N* = 9, 81.8%), diarrhea (*N* = 7, 63.6%), fatigue (*N* = 7, 63.6%), and respiratory signs (*N* = 5, 45.5%), including one patient who needed oxygen therapy. Three patients remained asymptomatic and COVID-19-free (including two under immunosuppressive treatments). Among the symptomatic patients, five showed atypical behaviors that we understood as idiosyncratic manifestations (e.g., irrepressible licking behavior). On day 14, only one patient with respiratory dysfunction still had a positive RT-PCR SARS-CoV-2 test. Conclusions: Organizing a COVID+ unit for patients with autism is realistic and requires close collaboration with infectologists. We believe that this initiative should be promoted to limit both the spread of the virus and the ostracism of patients with autism and challenging behaviors.

## 1. Introduction

COVID-19 is a multiorgan disease due to SARS-CoV-2 infection that has become pandemic in early 2020. The main symptoms are fever, dry cough, shortness of breath, fatigue, and mild to severe pneumopathy [1]. Gastrointestinal signs are infrequent but may be more common in elderly adults [2] and in youths [3]. A more severe outcome is associated with being older, being a male and having metabolic comorbidity, such as diabetes, hypertension, and obesity [1]. To date, very few reports beyond position papers have focused on mental health in the context of COVID-19 [4,5]. A search of the Medline database on 10 April 2020 yielded only one paper listing ten tips for handling autism spectrum disorder during restrictive measures to prevent the spread of COVID-19 [6]. Psychiatrists around the world have proposed specific risks for patients with chronic mental illnesses (such as schizophrenia, intellectual disabilities, and autism). First, patients with chronic mental illnesses, including individuals with autism, may be more prone to experiencing adverse consequences of quarantine [4]. Second, as with all vulnerable individuals, they are prone to abuse and ostracism, which, in the context of tensions regarding intensive care unit bed availability, might lead to discrimination with respect to access to care [7,8].

In the Paris area, SARS-CoV-2 was present as early as the winter of 2019, but the pandemic started in early March 2020, and restrictive measures were imposed beginning on 17 March 2020 [9]. As early as mid-March, the regional healthcare regulatory agency asked the neurobehavioral unit from the Pitié-Salpêtrière Hospital to convert the unit in a manner such that it could admit patients with autism, challenging behaviors (CBs), and COVID-19 if needed, for both psychiatric and medical care. Here, we report on this specific experience. We aimed to offer a detailed description of both the medical and behavioral manifestations of COVID-19 in individuals with autism and CBs, and to share with other practitioners how we attempted to address the spread of the virus and the use of protective equipment.

## 2. Experimental Section

### 2.1. Setting

The USIDATU (for Unité Sanitaire Interdépartementale d’Accueil Temporaire d’Urgence) is a neurodevelopmental unit dedicated to severe acute behavioral states and CBs in patients with autism and intellectual disability (ID). It is located in the Department of Child and Adolescent Psychiatry of the Pitié-Salpêtrière university hospital. The USIDATU seeks to provide both containment and supportive frameworks for patients [10,11]. The unit is composed of 14 single inpatient rooms and is divided into two sectors: the first for children and adolescents and the second for adults. Patients often show several psychiatric and medical comorbidities, including genetic [12] or rare syndromes [13]. It was originally modeled after a similar unit in the USA (http://www.kennedykrieger.org/patient-care/patient-care-programs/inpatient-programs/neurobehavioral-unit-nbu, 17 October 2014). As a constellation of causes can trigger and perpetuate CBs in individuals with autism, the same behaviors can be the expression of several causes, and a single cause can generate several CBs. The clinical approach first aims to formulate the maximum number of causal hypotheses and then to prioritize and treat them one by one according to a therapeutic intervention plan. The hypotheses are developmental, environmental, somatic, and psychiatric [10]. Treatment usually addresses all causes and includes a combination of behavioral measures following a psychoeducational approach, pain management using both analgesics and curative treatment (e.g., dental care in case of toothache), specific treatment targeting comorbid medical conditions (e.g., epilepsy), and psychotropic drugs targeting psychiatric comorbidity (e.g., antidepressants in cases of depression). In addition, specific attention is given to limit the iatrogenic effect of multiple prescriptions [14].

The Pitié-Salpêtrière hospital is the largest hospital in the Paris area and belongs to the Assistance Publique-Hôpitaux de Paris (AP-HP), the largest teaching hospital trust in Europe. As a result of its size and location, the AP-HP has been a major player in the regional COVID-19 response [15]. As soon as the pandemic started, the Department of Child and Adolescent Psychiatry of the Pitié-Salpêtrière hospital was asked to reorganize its inpatient units to offer a specific COVID-19 sector and to convert the neurobehavioral unit as a COVID-19 sector for individuals with autism and CBs who needed both psychiatric and COVID-19 medical care. We anticipated that controlling the spread of the virus in such patients would be especially challenging.

To achieve this objective, we organized a close cooperation with the Infectious and Tropical Diseases Department. This included a unique referral coordinator (AB), a clear monitoring prescription, shared decision-making in case of transfer in an intensive care unit, daily information regarding the stock of personal protective equipment, and the management of patients according to updated scientific knowledge. At the beginning of the pandemic, we also implemented two 3-h training sessions for 20 staff members to discuss how to limit the spread of the virus, how to wear and remove personal protective equipment, how to monitor COVID-19 patients (e.g., O2 saturation), and how to collect naso-pharyngeal samples for virus testing, which we usually do not practice in psychiatry.

However, managing the necessary COVID-19 safety precautions was quite challenging. Applying barrier measures such as using protective equipment, hand hygiene, and social distancing was impossible for these patients who exhibited such severe CBs. They were applied only by the staff. As long as the unit remained COVID-19+, the caregivers respected recommended dressing (non-valved FFP2 mask, non-water-resistant long-sleeved gown; plus gloves, single-use plastic apron worn over the gown, and goggles for proximity care). Patients’ meals were served in individual rooms and no longer in the common dining room. The unit’s bio-cleaning measures were maintained.

During the COVID-19 pandemic, the major goal of the USIDATU admission was medical stabilization for cases with significant comorbidities and risk factors for COVID-19. In other cases, neurobehavioral work continued as usual as possible with the major goal of reducing CBs, while ensuring medical safety regarding virus spread.

### 2.2. Participants and Ethics

The study was approved by the local ethical committee (Comité d’Ethique de Recherche—CER, Sorbonne Université, on 25 April 2020). All participants (or their legal representative) gave informed consent to participate. During the period of the pandemic (early March to mid-April), we had 22 patients who were positive for COVID-19 (virus test positive) or had probable COVID-19 (virus test negative, but symptoms compatible with COVID-19 combined with a close exposure to a COVID-19+ patient). Sixteen patients had autism. Given the number of inpatient beds of the department, patients could be contaminated on site or admitted because of psychiatric manifestations with COVID-19 comorbidity.

### 2.3. Collected Variables

Clinical variables were collected retrospectively using a grid that combined two sections: (1) psychiatric symptoms prior to COVID-19 (social interaction, stereotypies, restricted behaviors, language, challenging behaviors, autism severity based on DSM-5 criteria, general cognition, psychiatric comorbidity and medical comorbidity); and (2) physical signs that listed COVID-19 symptoms [16] based on the early literature mainly based on Asian experiences of the disease (see detailed list in Table 1). The diagnosis of COVID-19 was confirmed for all patients on the basis of a positive reverse transcriptase polymerase chain reaction (RT-PCR) assay from a nasopharyngeal swab (RealStar^®^, Hamburg, Germany) SARS-CoV-2 RT-PCR (Altona^®^, Hamburg, Germany)) [17]. Nasopharyngeal RT-PCR screening may have had false negative results, and we also collected COVID-19 diagnosis criteria based on the International Association of Infectious Diseases that includes prior exposure to SARS-CoV-2 microbiologically documented positive cases [18]. We also collected blood tests as requested by the infectious diseases team. To ensure COVID-19 diagnosis, we also serologically tested patients who had a negative RT-PCR screening for SARS-CoV-2 antibodies in early June using SARS-CoV-2 IgG ARCHITECT i System (Abbott^®^, Abbott Park, Illinois, US) commercial kits.

## 3. Results

**Figure 1:**
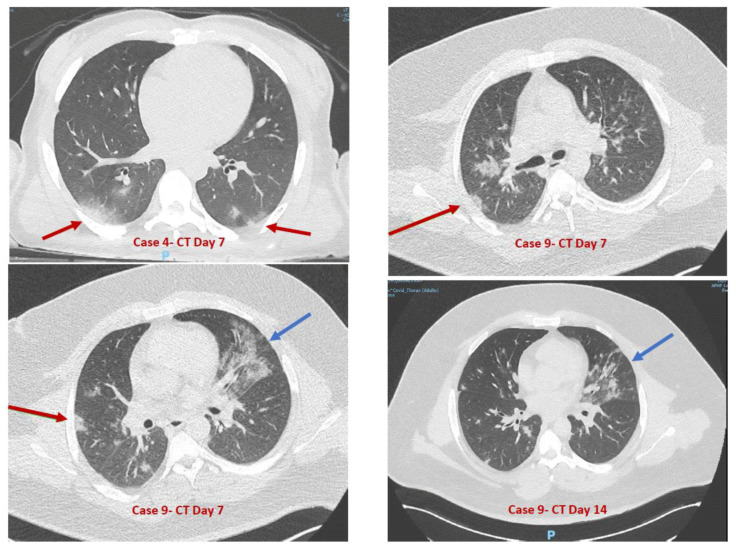
Chest CT (computerized tomography) scan of cases 4 and 9. The red arrow shows images of ground glass opacities; the blue arrow shows the area of secondary infection.

In total, we received 16 patients with autism and ID aged 12 to 43 years (mean age: 20.8 years) for neurobehavioral concern, for which COVID-19 was suspected. Four were females, and twelve were males. Eleven patients were already in-house when the pandemic hit the unit, and were nosocomially infected during the first week of the pandemic before the implementation of barrier measures against the SARS-CoV-2 virus. Five were novel admissions from the community. They were admitted because of CBs and sufficient medical complications from COVID-19 to require inpatient hospitalization. Based on nasopharyngeal RT-PCR or IgG searches, 11 cases were confirmed to have COVID-19 infections. Table 1 summarizes all patients’ clinical characteristics. The main COVID-19 symptoms included upper respiratory infection signs (e.g., rhinitis) (*N* = 9, 81.8%), diarrhea (*N* = 7, 63.6%), fatigue (*N* = 7, 63.6%), fever (*N* = 4, 36.4%), and respiratory signs (*N* = 5, 45.5%). One patient with a known history of epilepsy had a partial seizure that changed through a brief general seizure. Five showed atypical behaviors (see Table 1). In three patients, the course of diarrhea was notable and similar, with a relapse between day 7 and day 10 after an initial improvement. Table 2 summarizes the patients’ main paraclinical characteristics. Virus screening through RT-PCR was positive in nasopharyngeal samples for nine patients (81.8%). Three patients had CT scans, including case 9, who had several exams showing worsening respiratory conditions occurring on day 14, with imaging that was suggestive of bacterial pneumopathy (Figure 1). Only one patient among the four with respiratory dysfunction was positive for RT-PCR SARS-CoV-2 on day 14. Treatment was symptomatic in all cases and combined paracetamol for fever, nursing and restrictive measures to limit virus spread, oxygen supplementation, and antibiotics (amoxicillin-clavulanic acid 3 g-375 mg/day) to treat bacterial superinfection. Of note, there was no change in psychiatric medication except for one patient who received cyamemazine (100 mg/day) for 14 days. Similarly, we did not change immunosuppressive treatment in those who needed it (case 10: azhiatropine 50 mg/day; and case 15: imatinib 400 mg/day). Finally, three patients already in the unit were not infected and remained asymptomatic (including two under immunosuppressive treatments because of comorbid medical conditions).

## 4. Discussion

In this case series, patients with autism did show common signs of COVID-19, such as fever, fatigue, dyspnea, and diarrhea [1]. Additionally, the patient who still had a positive virus test through RT-PCR on day 14 had the most severe lesion with respiratory dysfunction [1]. Some points may be notable. First, the prevalence of diarrhea was higher and was similar to that described in pediatric or elderly populations [2,3]. This could be due to the rather young age of patients with diarrhea in this series (mean = 17.8 years). An alternative hypothesis is the known susceptibility of patients with autism to gastrointestinal disorders [20]. Second, some atypical symptoms needed to be understood as idiosyncratic manifestations in the specific sensory context of each patient. For example, we believe that the irrepressible licking behavior (case 1) and throwing away meals (case 5) may be understood as a consequence of anosmia/cacosmia or dysgeusia that constituted an acute change in the patient’s sensory functioning. Third, we had several nasopharyngeal swab negative RT-PCR results for SARS-CoV-2 virus tests (*N* = 7). Since SARS-CoV-2 IgG was positive in two patients in June, we estimated the proportion of false RT-PCR-negative patients to be approximately 20% (2/11). This has been reported [1]. However, we also want to acknowledge that nasal secretion collection was challenging in some patients. Fourth, we were anxious regarding our two patients who received immunosuppressive treatment. Surprisingly, they remained asymptomatic and had negative virus tests and IgG searches despite close and lengthy exposure to COVID-19 patients. Finally, two patients who had more restrictive measures before the pandemic due to high hetero-aggressive behaviors (cases 5 and 16) might have been contaminated through exposure to health care workers [1], before being transferred to our unit. For those who were not admitted primarily for medical stabilization, neurobehavioral work continued as usual as possible with the objective of reducing CBs. However, once fully dressed in recommended personal protective equipment all day, caregivers experienced discomfort and vulnerability, and strived to keep alive the relationship with the patient.

We are aware of the many limitations of such a case series. First, despite the large population served by the neurobehavioral unit, we only received 16 cases and 11 were COVID-19 confirmed cases. This low number of patients limits the generalization of the study. Second, we did not have a control group of the same age and sex. Interpretations proposed in the discussion section (e.g., frequency of diarrhea) need further research to be confirmed. Finally, we had no case that required the intensive care unit. We do not know whether specific issues would have occurred if we had to transfer a patient.

This small, recent experience in a specific unit also shows that organizing a COVID-19 unit for patients with autism is feasible if mental health politics support it. Unfortunately, this is not the case everywhere [7,8]. On day 14, most of the patients had negative PCR results, and no other transmission chain was detected to date inside the unit, meaning that we succeeded in limiting virus spread in an appropriate manner. To do so, we had to organize a close cooperation with the Infectious and Tropical Diseases Department, which implemented specific training to help staff members address the pandemic context. These measures also helped to limit the number of infections among staff members. The first week of the epidemic, we had 1 doctor and 5 nurses who were infected (representing 9% of the staff). Only one nurse was infected over the next 3 weeks. This is a remarkably low rate of transmission given the modes of transmission of SARS-CoV-2 virus (respiratory droplets, contact, but also airborne and fecal-oral transmission) and the challenging behaviors (e.g., spitting and spreading saliva, spreading stool) that many patients exhibit. We believe that these measures helped us avoid much higher rates of infection among health professionals seen in other psychiatric settings, where such measures have been delayed mainly because of a lack of supplies [7].

## 5. Conclusions

When infected by the SARS-CoV-2 virus, patients with autism and CBs present both common COVID-19 symptoms and idiosyncratic manifestations. Organizing a COVID+ unit for such patients is realistic and requires close collaboration with infectologists. We believe that this initiative should be promoted to limit both the spread of the virus and the ostracism of patients with autism.

## Figures and Tables

**Table 1:** Clinical characteristics of patients with autism and COVID-19.

			ASD Symptoms Prior COVID								COVID-19 Acute Symptoms (JX; Y Days) **						
	Sex	Age	Social Interaction	Stereotypic, Restricted Behaviors	Challenging Behaviors	Language	Autism * Severity	General Cognition	Psychiatric Comorbidity	Medical Comorbidity	Fever	Oral-Facial	Respiratory	Fatigue	Gastro-Intestinal	Brain	Atypical Behaviors
Case 1	F	23	Limited social-emotional reciprocity and nonverbal communicative behaviors	Touching people, page flipping	No	Few spoken words	Level 2	Moderate ID	No	Class I obesity (BMI = 30)	No	Possible dysgeusia (J1; 5); epistaxis (J4; <15 min)	No	No	No	No	Irrepressible licking behavior (J1; 5), puts her index finger to her mouth (J1; 5)
Case 2	F	26	Limited social-emotional reciprocity, no nonverbal communicative behaviors	Strolling	SIB	Nonverbal	Level 3	Profound ID	Bipolar disorder	Recurrent urinary tract infections, chronic constipation	No	Rhinitis (J1; 7)	No	No	No	No	Crying, irritability, head blows in the walls (J3; 4)
Case 3	F	43	Limited social-emotional reciprocity and nonverbal communicative behaviors	Body swings, hand clapping	Mild SIB, mild hetero-aggressivity	Short sentences echolalia	Level 3	Moderate ID	Bipolar disorder	Prematurity, repetitive infections (urine, skin, breast), constipation, tooth decay	Yes (J1; 1)	Rhinitis (J2; a few days)	Dry cough (J5; 3)	Yes (J6; 10)	Diarrhea (J3; a few days)	No	Increased hetero-aggressivity and sleep disorders the week before (associated with an urinary tract infection 5 days before fever)
Case 4	M	24	Limited social-emotional reciprocity and nonverbal communicative behaviors	Invasive food search, echopraxia	Mild SIB, hetero-aggressivity	Few spoken words, echolalia	Level 3	Severe ID	No	Epilepsy, neuropathic pain, gastroparesis surgery	Yes (J3; 1)	Rhinitis (J1; 12)	Productive cough, polypnea (J4; 5)	Yes (J3; 10)	Diarrhea (J3; 1)	No	No
Case 5	M	16	Limited social-emotional reciprocity, limited facial expressions, some interest in maintaining verbal relationships	Pseudo OCD, echolalia, smells food before eating	Moderate hetero-aggressivity	Functional language	Level 2	Mild ID	Bipolar disorder	Meningitis, horizontal nystagmus	No	Anosmia, dysgeusia (J1; <10)	No	No	No	No	Threw away his meal trays (J1; 3)
Case 6	M	16	Limited social-emotional reciprocity and nonverbal communicative behaviors, significant autism withdrawal	Puts objects in his ears and nose	Hetero-aggressivity	Nonverbal few Makaton signs	Level 3	Severe ID	Major depressive disorder	Epilepsy, Behcet’s syndrome, chronic constipation	Yes (J1; 1)	Rhinitis (J1; 6)	No	Yes (J1; 4)	Diarrhea (J2; 3 then J9; 2)	No	No
Case 7	M	20	Limited social-emotional reciprocity and nonverbal communicative behaviors	Upper body motor stereotypies, pseudo OCD	SIB	Nonverbal few Makaton signs	Level 3	Severe ID	Major depressive disorder with catatonic features	Pharmacoresistant epilepsy, gastritis, severe chronic constipation	No	No	Dry cough (J1; 5)	Yes (J3; 10)	Diarrhea (J3; 3 then J8; 6)	No	Stool spread during episodes of diarrhea; increase in SIB (J6; 10)
Case 8	M	15	Limited social-emotional reciprocity, no nonverbal communicative behaviors	Strolling, pica, spitting and spreading saliva	No	Nonverbal	Level 3	Severe ID	No	Epilepsy, gastric mastocytosis, delayed puberty	No	Rhinitis (J1; 3)	No	Yes (J1; 3)	Diarrhea (J4; 4 then J10; 5)	No	No
Case 9	M	28	Limited social-emotional reciprocity and nonverbal communicative behaviors	No	No	Nonverbal	Level 3	Severe ID	No	Epilepsy, class III obesity (BMI = 55), HBP	Yes (J1; <36 h)	No	Dry cough (J1; 2), polypnea (J1; >9 days) needing oxygen	No	No	Epilepsy (J7, 1 episode)	No
Case 10	M	16	Limited social-emotional reciprocity and nonverbal communicative behaviors	Motor stereotypies, OCD	Moderate hetero-aggressivity	Few sentences echolalia	Level 3	Severe ID	No	Seronegative autoimmune encephalitis (responsive to immuno-suppressive drug)	No	No	No	No	No	No	No
Case 11	M	16	Limited social-emotional reciprocity and nonverbal communicative behaviors	Flapping, pseudo OCD	Mild SIB, hetero-aggressivity	Nonverbal few Makaton signs	Level 3	Severe ID	Major depressive disorder	Epilepsy, gastric ulcer, chronic hives, constipation	No	No	Dry cough (J1; 3)	No	No	No	No
Case 12	F	13	Limited social-emotional reciprocity and nonverbal communicative behaviors	Body swings, tiptoeing	No	Few spoken words	Level 3	Severe ID	No	Severe constipation	No	Rhinitis (J1; 2)	No	Yes (J1; 2)	Diarrhea (J3; 7)	No	No
Case 13	M	12	Limited social-emotional reciprocity, some effective nonverbal communicative behaviors	Strolling	Severe SIB	Nonverbal sign language	Level 2	Moderate ID	No	CHARGE syndrome, unilateral cecity, deafness, anosmia	No	No	No	No	No	No	No
Case 14	M	16	Limited social-emotional reciprocity and nonverbal communicative behaviors	Attention search	Sexual behaviors	Functional language	Level 3	Severe ID	Bipolar disorder, anxiety disorder	Migraine, hiatal hernia, esophagitis, gastritis, class I obesity (BMI = 31), inflammatory bowel disease	No	Rhinitis (J1; 4)	No	Yes (J1; 4)	No	No	No
Case 15	M	14	Limited social-emotional reciprocity, limited facial expressions, difficulties adjusting behavior to suit various social contexts, some interest in peers	Attention search	No	Functional language	Level 1	Moderate ID	No	Chronic myeloid leukemia (immuno-suppressive drug)	No	No	No	No	No	No	No
Case 16	M	34	Limited social-emotional reciprocity and nonverbal communicative behaviors	Wandering, vocalizations	Punctual hetero-aggressivity	Few spoken words	Level 3	Severe ID	Anxiety disorder	PRODH gene deletion, Ehlers-Danlos syndrome, restless legs syndrome, gastritis, constipation	No	No	No	No	Diarrhea (J1; 5)	No	No

* According to DSM-5 levels of support: Level 1 (Requiring support); Level 2 (Requiring substantial support); Level 3 (Requiring very substantial support); ** X: day of apparition of the symptom; Y: duration of the symptom (in days); ASD: autism spectrum disorder; BMI: body mass index; CHARGE: coloboma, heart defects, atresia choanae, growth retardation, genital abnormalities, ear abnormalities; COVID: coronavirus disease; HBP: high blood pressure; ID: intellectual disability; OCD: obsessive-compulsive disorder; PRODH: proline dehydrogenase; SIB: self-injurious behaviors.

**Table 2:** Paraclinical characteristics of patients with autism and COVID-19.

	Close Exposition to a PCR+ COVID-19 Patient *	SARS-CoV2 PCR Screening	SARS-CoV2 IgG Screening	Chest CT Scan	Other Imaging	Blood Test	COVID-19
Case 1	Yes	Positive	Not performed	No	No	At Day 2: Neutropenia (1.78 × 10⁹/L), Aspartate AminoTransferase elevation (×2 N)	Yes
Case 2	Yes	Positive	Not performed	No	No	At Day 4: Anemia (11.2 g/dL), Elevated C-Reactive Protein (8.78 mg/L)	Yes
Case 3	Yes	Positive	Not performed	No	No	At Day 1: Elevated C-Reactive Protein (51 mg/L)	Yes
Case 4	Yes	Positive	Not performed	Yes	No	At Day 6: Anemia (12.9 g/dL), Neutropenia (1.58 × 10⁹/L), Elevated C-Reactive Protein (9.59 mg/L)	Yes
Case 5	No (psychiatric secure room)	Positive	Not performed	No	No	Not performed as paucisymptomatic	Yes
Case 6	Yes	Positive	Not performed	No	No	At Day 7: Elevated C-Reactive Protein (6.07 mg/L), Lactate Dehydrogenase elevation (×1.5 N)	Yes
Case 7	Yes	Positive	Not performed	Yes	No	At Day 7: Anemia (12.7 g/dL), Neutropenia (1.75 × 10⁹/L), Elevated C-Reactive Protein (6.04 mg/L)	Yes
Case 8	Yes	Positive	Not performed	No	No	At Day 7: Neutropenia (1.70 × 10⁹/L), Lactate Dehydrogenase elevation (×1.1 N)	Yes
Case 9	Yes	Negative	Positive	Yes	No	At Day 6: Elevated C-Reactive Protein (22.4 mg/L), Aspartate AminoTransferase elevation (×1.5 N)	Yes
Case 10	Yes	Negative	Negative	No	No	Under immunosuppressive regimen: Anemia (11.7 g/dL), Leukopenia (3.51 × 10⁹/L), Lymphopenia (0.76 × 10⁹/L)	No
Case 11	Yes	Negative	Negative	No	No	At Day 9: Lymphocytosis (4.46 × 10⁹/L)	No
Case 12	Yes	Negative	Negative	No	No	Not performed as paucisymptomatic	No
Case 13	Yes	Negative	Negative	No	No	Not performed as asymptomatic	No
Case 14	Yes	Negative	Positive	No	No	Not performed as paucisymptomatic	Yes
Case 15	Yes	Negative	Negative	No	No	Under immunosuppressive regimen: Anemia (12.0 g/dL), Lactate Dehydrogenase elevation (×1.1 N)	No
Case 16	No (psychiatric secure room)	Positive	Not performed	No	No	Not performed	Yes

* Person who had prolonged (>15 min) direct face-to-face contact within 1 m with a confirmed case, shared the same hospital room, lived in the same household, or shared any leisure or professional activity in close proximity with a confirmed case, or travelled together with a COVID-19 case in any kind of conveyance, without appropriate individual protection equipment. Healthcare personnel who treated a confirmed case without wearing appropriate personal protective equipment or with an identified breach [19].

## References

[1] Xie M., Chen Q. (2020). Insight into 2019 novel coronavirus-An updated interim review and lessons from SARS-CoV and MERS-CoV. Int. J. Infect. Dis..

[2] Choi S.H., Kim H.W., Kang J.M., Kim D.H., Cho E.Y. (2020). Epidemiology and clinical features of coronavirus disease 2019 in children. Clin. Exp. Pediatr..

[3] Cheung K.S., Hung I.F., Chan P.P., Lung K.C., Tso E., Liu R., Ng Y.Y., Chu M.Y., Chung T.W.H., Tam A.R. (2020). Gastrointestinal Manifestations of SARS-CoV-2 Infection and Virus Load in Fecal Samples From a Hong Kong Cohort: Systematic Review and Meta-analysis. Gastroenterology.

[4] Brooks S.K., Webster R.K., Smith L.E., Woodland L., Wessely S., Greenberg N., Rubin G.J. (2020). The psychological impact of quarantine and how to reduce it: Rapid review of the evidence. Lancet.

[5] Holmes E.A., O’Connor R.C., Perry V.H., Tracey I., Wessely S., Arseneault L., Ballard C., Christensen H., Silver R.C., Everall P. (2020). Multidisciplinary research priorities for the COVID-19 pandemic: A call for action for mental health science. Lancet Psychiatry.

[6] Narzisi A. (2020). Handle the Autism Spectrum Condition During Coronavirus (COVID-19) Stay At Home period: Ten Tips for Helping Parents and Caregivers of Young Children. Brain Sci..

[7] Arango C. (2020). Lessons Learned From the Coronavirus Health Crisis in Madrid, Spain: How COVID-19 Has Changed Our Lives in the Last 2 Weeks. Biol. Psychiatry.

[8] Chevance A., Gourion D., Hoertel N., Llorca P.M., Thomas P., Bocher R., Moro M.-R., Laprévote V., Benyamina A., Fossati P. (2020). Ensuring mental health care during the SARS-CoV-2 epidemic in France: A narrative review. Encephale.

[9] Deslandes A., Berti V., Tandjaoui-Lambotte Y., Alloui C., Carbonnelle E., Zahar J.R., Brichler S., Cohen Y. (2020). SARS-CoV-2 was already spreading in France in late December 2019. Int. J. Antimicrob. Agents.

[10] Guinchat V., Cravero C., Diaz L., Perisse D., Xavier J., Amiet C., Gourfinkel-An I., Bodeau N., Wachtel L., Cohen D. (2015). Acute behavioral crises in psychiatric inpatients with autism spectrum disorder (ASD): Recognition of concomitant medical or non-ASD psychiatric conditions predicts enhanced improvement. Res. Dev. Disabil..

[11] Levine J., Cohen D., Herman C., Verloes A., Guinchat V., Diaz L., Cravero C., Mandel A., Gozes I. (2019). Developmental Phenotype of the Rare Case of DJ Caused by a Unique ADNP Gene De Novo Mutation. J. Mol. Neurosci..

[12] Cravero C., Guinchat V., Xavier J., Meunier C., Diaz L., Mignot C., Doummar D., Chantot-Bastaraud S., Consoli A., Cohen D. (2017). Management of Severe Developmental Regression in an Autistic Child with a 1q21.3 Microdeletion and Self-Injurious Blindness. Case Rep. Psychiatry.

[13] Cravero C., Guinchat V., Barete S., Consoli A. (2016). Cornelia de Lange and Ehlers-Danlos: Comorbidity of two rare syndromes. BMJ Case Rep..

[14] Guinchat V., Gallagher C.B.A., Bulteau C., Cohen D., Michaud J.L. (2020). Multidisciplinary Treatment Plan for Challenging Behaviors in Neurodevelopmental Disorders. Handbook of Neurocognitive Development: Disorders and Disabilities.

[15] COVID19-APHP Group (2020). Assistance Publique-Hopitaux de Paris’ response to the COVID-19 pandemic. Lancet.

[16] The Royal College of Physicians National Early Warning Score (NEWS) 2|RCP London. https://www.rcplondon.ac.uk/projects/outputs/national-early-warning-score-news-2.

[17] Corman V.M., Landt O., Kaiser M., Molenkamp R., Meijer A., Chu D.K., Bleicker T., Brünink S., Schneider J., Schmidt M.L. (2020). Detection of 2019 novel coronavirus (2019-nCoV) by real-time RT-PCR. Euro. Surveill..

[18] Santé Publique France (2020). Infection au Nouveau Coronavirus (SARS-CoV-2), COVID-19, France et Monde. https://www.santepubliquefrance.fr/maladies-et-traumatismes/maladies-et-infections-respiratoires/infection-a-coronavirus/articles/infection-au-nouveau-coronavirus-sars-cov-2-covid-19-france-et-monde.

[19] Stoecklin S.B., Rolland P., Silue Y., Mailles A., Campese C., Simondon A., Mechain M., Meurice L., Nguyen M., Bassi C. (2020). First cases of coronavirus disease 2019 (COVID-19) in France: Surveillance, investigations and control measures, January 2020. Euro. Surveill..

[20] Hyman S.L., Levy S.E., Myers S.M. (2020). Identification, Evaluation, and Management of Children With Autism Spectrum Disorder. Pediatrics.

